# Relationship between high fructose corn syrup sweetened drinks, diet soft drinks, and serum sodium: NHANES 2003–2006

**DOI:** 10.1186/s12937-022-00832-7

**Published:** 2022-12-29

**Authors:** Mingxi Li, Weijun Gong, Shidong Wang, Zhe Li

**Affiliations:** 1 Department of Traditional Chinese Medicine, Beijing Rehabilitation Hospital, Capital Medical University, Xixiazhuang Badachu, Shijingshan District, Beijing, 100144 China; 2 Department of Endocrinology, Dongzhimen Hospital, Beijing University of Chinese Medicine, No.5 Haiyuncang, Dongcheng District, Beijing, 100700 China

**Keywords:** A cross-sectional study, Serum sodium, High fructose corn syrup sweetened beverages, Artificially sweetened beverages, Fructose and hypertension, Regression analysis

## Abstract

**Background:**

Consumption of high fructose corn syrup sweetened drinks and diet soft drinks has increased in the United States. However, the relationship between the intake of high fructose corn syrup sweetened drinks and diet soft drinks, and serum sodium has been scarcely studied. Our objective is to evaluate the relation between intake of high fructose corn syrup sweetened drinks and diet soft drinks, and serum sodium, and explore the possible effect modifiers in a nationally representative sample of adults from the United States.

**Methods:**

We conducted a cross-sectional study using data from the National Health and Nutrition Examination Survey 2003–2006. The study participants included 6989 adults aged ≥18 years. Using survey-weighted generalized linear regression analyses, we investigated the relationship between high fructose corn syrup sweetened drink, diet soft drink consumption, and serum sodium. Consumption of high fructose corn syrup sweetened drinks and diet soft drinks was evaluated through a food-frequency questionnaire.

**Results:**

Serum sodium levels increased as high fructose corn syrup sweetened drink intake increased. Serum sodium levels were higher in participants in the highest high fructose corn syrup sweetened drink consumption quantile, compared with those in the lowest high fructose corn syrup sweetened drink intake quantile (*p* = 0.020). The multivariate betas for serum sodium, according to the corresponding high fructose corn syrup sweetened drink intake quantiles, were 0.16, 0.19, and 0.21, respectively (*P* for trend = 0.051). We found no relationship between diet soft drink consumption and serum sodium after adjustment of confounding. (multivariate *P* > 0.05).

**Conclusion:**

There was a a step-wise increase in serum sodium concentration with increasing consumption of HFCS sweetened beverages. Even moderate HFCS sweetened soft drink intake was associated with an elevated serum sodium level - a risk factor for hypertension.

## Introduction

Over the past decades, consumption of high fructose corn syrup (HFCS) sweetened drinks has increased greatly and constituted a significant component of energy intake globally [1–4]. One-half of US adults consume ≥1 HFCS beverage daily, representing 6.5% of total energy intake [5]. Diet soft drinks are marketed as healthier and are suggested as alternatives to HFCS sweetened beverages [6, 7]. Several studies have shown that consumption of diet soft drinks has increased dramatically from about 3% of adults in 1965 to about 20% of adults today [6, 7]. A growing body of observational evidence suggests that soft drink consumption may parallel the increase in blood pressure [8–13].

A recently published article by DeChristopheret al. reported that larger consumption of soft drinks is positively associated with coronary heart disease among African Americans, possibly due to fructose malabsorption, dysbiosis, and gut formation of advanced glycation end-products [14]. There are some possible biological mechanisms may explain the association between HFCS sweetened drinks consumption and the risk of hypertension. HFCS sweetened drinks contain high fructose; and high-fructose consumption disrupts the intestinal barrier, increases gut permeability, causes profound gut microbiota dysbiosis, and dysregulates T-lymphocytes [15–18]. With decreased bacterial diversity, the overgrowth of some commensals, and concomitant suppression of others, gut microbiota dysbiosis leads to an altered ratio of short-chain fatty acids (SCFA) [19]. Intriguingly, fecal SCFA concentrations in humans have been associated with higher blood pressure, while SCFA-producing microbiota is often associated with lower blood pressure [20]. A growing body of research has shown that gut microbiota dysbiosis is associated with high blood pressure [21, 22].

Some possible metabolic mechanisms might also explain the positive associations between HFCS sweetened drink consumption and the risk of hypertension [15, 23, 24]. HFCS sweetened drinks are the greatest source of fructose-containing sugars in the diet, and the effect of HFCS sweetened drinks on cardiometabolic diseases is mainly from fructose [24]. Fructose can also cause intracellular adenosine triphosphate depletion, nucleotide turnover, and the generation of uric acid, which may lead to an increase in insulin resistance and further diabetes and cardiovascular disease [24–26]. HFCS sweetened drinks have been suggested to increase serum uric acid concentrations, insulin resistance, and obesity, which activates the renin-angiotensin system [23, 27], .and leads to acute endothelial dysfunction, renal microvascular alteration, and chronic sodium retention [28]. Sodium, the main extracellular cation, has long been considered the pivotal factor in hypertension [29]. Numerous studies show an adverse effect of a surfeit of sodium on arterial pressure [30–32]. Meanwhile, a recent five-year cohort study in Japan showed that serum sodium concentration could predict the development of hypertension [33].

Some studies reported that diet soft drink consumption is positively associated with hypertension [9, 10]. However, such a relationship can be affected by multiple factors such as body weight and poor health [9]. Previous studies have also reported a significant relationship between diet soda consumption and metabolic syndrome [34, 35]. These associations are generally speculated to be the result of residual confounding by other dietary behaviors, lifestyle factors, or demographic characteristics [36]. Meanwhile, the relationship between the intake of HFCS sweetened drinks and diet soft drinks, and serum sodium has been scarcely studied. It remains unclear whether there is a pre-metabolic mechanism between HFCS sweetened soft drink consumption and serum sodium. We hypothesized that HFCS sweetened drink consumption is positively associated with serum sodium levels, but that diet soft drink intake is not. We thus conducted this cross-sectional study to evaluate the relationship between HFCS sweetened drink intake, diet soft drink intake, and serum sodium.

## Materials and methods

### Study population

The National Health and Nutrition Examination Survey (NHANES) conducted during 2003–2006 (2003–2004 cycle and 2005–2006 cycle) is a nationally representative adult survey in the United States, which collects data from survey participants through household interviews, standardized physical examinations, and laboratory tests in mobile examination centers (MEC) [37]. Detailed information on the NHANES procedures is available in the literature [38].

The present study used NHANES data including adults aged ≥18 years (*N* = 11,183) collected from the 2003–2006 cycles. Among the 20,470 participants (10,420 men and 10,050 women), 5396 had complete data on both HFCS sweetened drink intake and serum sodium, and 2731 had complete data on both diet soft drink intake and serum sodium. Totally, 6989 participants were included in the analysis. Approval was obtained from the National Center for Health Statistics Research Ethics Review Board, and all participants provided written informed consent.

### Serum sodium measurement

We obtained serum sodium concentrations from the NHANES 2003–2006 laboratory files. The serum sodium levels were determined by Beckman LX system utilizing indirect (or diluted) I.S.E. methodology. More details of the measurement methods and quality control procedures have been described elsewhere [38]. Serum sodium values were expressed as micromoles per liter (mmol/L).

### Assessment of HFCS drink and diet soft drink intake

During the household interview, intakes of HFCS sweetened drinks and diet soft drinks were determined based on the responses of study participants to a food-frequency questionnaire, which was used to evaluate their average intake in the past month (can only be obtained during 2003–2004 cycle and 2005–2005 cycle). The food frequency questionnaire defined HFCS sweetened drinks as soda, pop, or other soft drinks that contain HFCS. Diet soft drinks were defined as soda, pop, or other soft drinks diet or sugar-free. Collecting information on food frequency during household interviews has been proven to be a reliable and valid method for assessing usual consumption [39, 40].

### Assessment of covariates

Data on total energy intake, dietary sodium intake, and total moisture intake were obtained from the total nutrient intakes file (second-day dietary interview), which contains a summary of an individual’s nutrition from all foods and beverages provided on the dietary recall. Information about age, sex, race, and education was obtained from demographic data. Information about anthropometric measurements (including height and weight) was obtained from examination data. Information on the medical history of hypertension was obtained from questionnaire data. Serum potassium, serum sodium, serum chloride, serum uric acid, and osmolality levels were obtained from laboratory data. Family income-to-poverty ratio was categorized into two groups: < 130% and ≥ 130%. Physical activity during the past month was assessed by using a questionnaire in NHANES 2003–2006. Based on the 2018 Physical Activity Guidelines for Americans, respondents who engaged in moderate-intensity aerobic activity for 150 min/week or vigorous-intensity aerobic activity for 75 min/week, or an equivalent combination of both (1 min of vigorous-intensity physical activity is equivalent to 2 min of moderate intensity physical activity) were defined as meeting the guidelines [41]. In our analysis, physical activity was categorized into three levels: sufficiently active, insufficiently active, and inactive. Sufficiently active was defined as a moderate-intensity aerobic activity for 150 min/week or vigorous-intensity aerobic activity for 75 min/week, or an equivalent combination of both. Insufficiently active was defined as some aerobic activity but not enough to meet the guidelines (10–149 min/week). Inactive was defined as some physical activity (< 10 min/week) or no physical activity reported [41]. This classification of physical activity has been used in previous studies [42].

Body mass index (BMI) was calculated by dividing weight (in kilograms) by height squared (in meters). Estimated glomerular filtration rate (eGFR) was calculated by using the Chronic Kidney Disease Epidemiology Collaboration (CKD-EPI) equation [43]:

GFR (ml/minute per 1.73 m^2^) =141 × min (Scr/κ, 1)^α^ × max(Scr/κ, 1) ^-1.209^ × 0.993^Age^ × 1.018 [if female] × 1.159 [if black], where Scr is serum creatinine, κ is 0.7 for women and 0.9 for men, α is − 0.329 for females and − 0.411 for males, min indicates the minimum of Scr/κ or 1, and max indicates the maximum of Scr/κ or 1.

### Statistical analysis

All statistical analyses were performed using R software (R Foundation for Statistical Computing, Vienna, Austria, Version 3.6.3) and the “survey” package (e.g., svymean and svyglm), which considers sampling weights (4-year MEC exam weight), clustering, and stratification of the complex survey design [44]. Participants were grouped according to quantiles of HFCS drink intake and diet soft drink intake. Continuous variables were reported as weighted means and standard errors, while categorical variables were reported as weighted proportions. Comparisons between HFCS sweetened drink quantile groups and diet soft drink intake quantile groups were made by using the t-test (continuous variables), wald-test (categorical variables), one-way ANOVA test (normal distribution), or Kruskal-Wallis H test (skewed distribution).

We used survey-weighted generalized linear regression modeling to examine the relationship between HFCS sweetened drink and diet soft drink intake, and serum sodium. HFCS sweetened and diet soft drink consumption was categorized into quantiles. The serum sodium level is closely controlled by water homeostasis, which is mediated by thirst, arginine vasopressin, and the kidneys [45]. Meanwhile, people who drink HFCS sweetened drinks and diet soft drinks tend to also consume high dietary sodium [46]. Thus, we included osmolality, eGFR, total sodium intake, and total energy intake as covariates in the analyses. In short, variables (physical activity, history of hypertension, diuretics use, BMI, serum potassium, serum chloride, osmolality, eGFR, and intake of dietary sodium, total moisture and total energy) thought to be confounders based on existing literature [29, 47–50] and clinical judgment were included. Multivariate survey-weighted generalized linear regression models were adjusted for age, sex, race, education, poverty income ratio, physical activity, history of hypertension, diuretics use, BMI, serum potassium, serum chloride, osmolality, eGFR, and intakes of dietary sodium, total moisture, and total energy. A *P*-value for trend was obtained by entering the quantile HFCS sweetened drinks and diet soft drinks intakes variables as continuous variables, and rerunning the corresponding survey-weighted generalized linear regression models.

Prespecified subgroup analyses were conducted according to age, sex, race, BMI, eGFR, poverty income ratio, and activity status. Tests of interaction were run for all subgroups. For all difference estimates (betas), 95% confidence intervals (95% CIs) were calculated. All statistical tests were two-sided, and statistical significance was set at *p* ≤ 0.05.

## Results

### Study participants and baseline characteristics

**Fig. 1:**
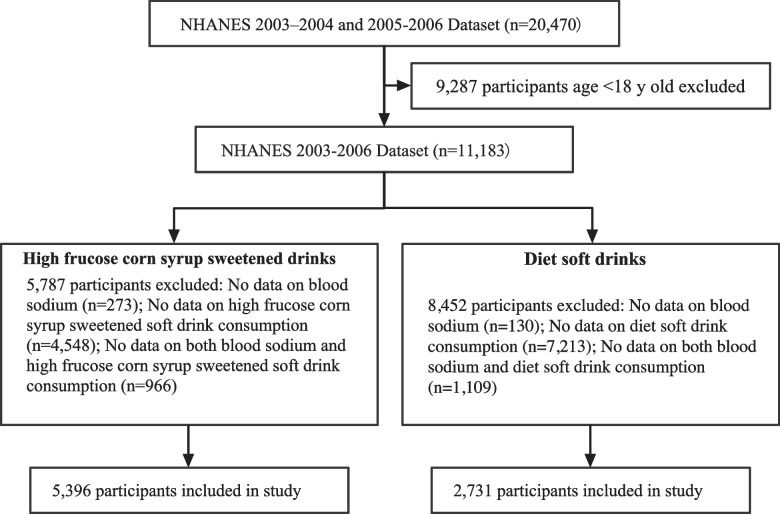
Flowchart of participants selection

The flow chart of the research is shown in Fig. 1. In total, 5396 participants who drink HFCS sweetened drinks and 2731 who drink soft drinks were included in our final analysis. The demographic and clinical characteristics of all participants according to HFCS sweetened drink intake quantile are listed in Table 1. The weighted population’s mean (SE) age was 45.40 (0.46) years, 50.50% of the population were men, and the weighted mean (SE) serum sodium was 139.09 (0.07) mmol/L. The relevant characteristics according to diet soft drink intake quantile are shown in Table 2. The weighted population’s mean (SE) age was 48.98 (0.49) years, 40.36% of the population were men, and the weighted mean (SE) serum sodium was 139.07 (0.09) mmol/L. BMI, eGFR, dietary sodium intake, and total energy intake tended to increase with increasing intake of HFCS sweetened or diet drinks, however, age tended to decrease.

**Table 1:** Baseline characteristics of participants (HFCS sweetened drinks)^a^

Characteristic	Total (***n*** = 5396)	HFCS sweetened soft drink intake quantiles (servings/day)^**1**^				***P***-value^**2**^
		Quantile1 (***n*** = 1470)	Quantile2 (***n*** = 1259)	Quantile3 (***n*** = 1279)	Quantile4 (***n*** = 1388)	
Age, years	45.40 ± 0.46	52.47 ± 0.87	46.60 ± 0.66	42.63 ± 0.50	39.58 ± 0.53	< 0.001
Sex, n (%)						< 0.001
Male	2607 (50.50)	608 (39.97)	578 (47.08)	671 (55.88)	750 (59.43)	
Female	2789 (49.50)	862 (60.03)	681 (52.92)	608 (44.12)	638 (40.57)	
Race, n (%)						< 0.001
Non-Hispanic White	2626 (70.50)	838 (74.61)	601 (69.21)	544 (66.40)	643 (71.02)	
Non-Hispanic Black	1202 (11.84)	241 (8.87)	284 (12.54)	318 (13.90)	359 (12.44)	
Hispanic	171 (3.65)	47 (3.62)	36 (3.11)	46 (4.69)	42 (3.25)	
Mexican American	1169 (8.27)	265 (5.88)	279 (8.21)	331 (10.50)	294 (8.79)	
Other race	228 (5.74)	79 (7.02)	59 (6.92)	40 (4.51)	50 (4.50)	
Education, n (%)						< 0.001
Less than high school	1400 (18.48)	374 (16.26)	311 (16.49)	328 (18.84)	387 (22.27)	
High school graduate	1242 (27.82)	320 (24.33)	289 (25.58)	293 (27.91)	340 (33.40)	
Some college or AA degree	1315 (30.46)	364 (28.39)	329 (31.11)	321 (32.36)	301 (33.36)	
College graduate or above	866 (23.17)	325 (30.85)	233 (26.73)	181 (20.88)	127 (13.98)	
Poverty income ratio, n (%)						0.016
< 130%	1616 (21.50)	392 (18.48)	346 (19.01)	386 (21.42)	492 (26.70)	
≥ 130%	3516 (78.50)	996 (81.52)	854 (80.99)	828 (78.58)	838 (73.30)	
Physical activity, n (%)						0.2
Inactive	176 (5.22)	35 (3.89)	41 (4.63)	40 (6.43)	60 (6.10)	
Insufficiently active	1621 (48.67)	469 (46.72)	343 (48.29)	365 (48.78)	444 (50.89)	
Sufficiently active	1529 (46.11)	445 (49.40)	330 (47.08)	318 (44.79)	436 (43.01)	
History of hypertension, n (%)						< 0.001
No	3235 (62.14)	768 (54.57)	733 (62.70)	818 (66.37)	916 (65.67)	
Yes	2158 (37.86)	701 (45.43)	525 (37.30)	460 (33.63)	472 (34.33)	
Diuretic use, n (%)						< 0.001
No	4783 (90.27)	1216 (84.12)	1120 (91.65)	1162 (91.81)	1285 (93.97)	
Yes	613 (9.73)	254 (15.88)	139 (8.35)	117 (8.19)	103 (6.03)	
BMI, kg/m^2^	27.89 ± 0.17	27.16 ± 0.25	27.84 ± 0.29	27.90 ± 0.22	28.67 ± 0.29	0.005
Na, mmol/L	139.09 ± 0.07	138.92 ± 0.10	139.20 ± 0.09	139.08 ± 0.10	139.16 ± 0.09	0.3
K, mmol/L	3.98 ± 0.01	4.00 ± 0.02	3.98 ± 0.01	3.99 ± 0.01	3.95 ± 0.01	0.023
Cl, mmol/L	103.63 ± 0.07	103.28 ± 0.11	103.78 ± 0.09	103.75 ± 0.09	103.77 ± 0.13	0.006
UA, mmol/L	320.46 ± 1.57	312.51 ± 3.09	315.46 ± 2.56	325.30 ± 2.91	327.41 ± 2.71	< 0.001
Osmolality, mmol/kg	277.36 ± 0.15	277.56 ± 0.26	277.66 ± 0.20	277.21 ± 0.21	277.03 ± 0.19	0.037
eGFR, mL/min/1.73m^2^	91.99 ± 0.57	85.50 ± 1.13	91.60 ± 0.92	93.75 ± 0.70	97.37 ± 0.62	< 0.001
HFCS sweetened soft drink intake, servings/day	0.78 ± 0.04	0.02 ± 0.001	0.14 ± 0.002	0.54 ± 0.006	2.33 ± 0.06	< 0.001
Dietary sodium intake, mg/day	3406.6 ± 41.97	3126.90 ± 59.16	3354.06 ± 65.13	3541.58 ± 78.55	3.620.39 ± 64.46	< 0.001
Total moisture intake, g/day	2251.20 ± 31.63	2296.55 ± 63.55	2191.80 ± 47.53	2261.70 ± 46.40	2247.79 ± 30.29	0.140
Total energy intake, kcal/d	2135.10 ± 21.56	1938.31 ± 32.41	2051.48 ± 39.41	2229.07 ± 48.13	2327.41 ± 29.41	< 0.001

*Abbreviations*: *BMI* Body mass index, *CI* Confidence interval, *eGFR* Estimated glomerular filtration rate, *HFCS* High fructose corn syrup, *UA* Uric acid

^1^Values are presented as mean ± SE for continuous variables and unweighted numbers (weighted%) for categorical variables

^2^*P*-values are for the difference between the four HFCS sweetened soft drink quantiles. For the determination of p-values, svy-t-tests were used for continuous variables, and svy-wald-tests were used for categorical variables

^a^Data are presented incorporating sample weights and adjusted for clusters and strata of the complex sample design of the National Health and Nutrition Examination Survey (2003–2004 cycle and 2005–2006 cycle)

**Table 2:** Baseline characteristics of participants (Diet soft drinks)^a^

Characteristic	Total (***n*** = 2731)	Diet soft drink intake quantiles (servings/day)^**1**^				***P***-value^**2**^
		Quantile1 (***n*** = 567)	Quantile2 (***n*** = 652)	Quantile3 (***n*** = 718)	Quantile4 (***n*** = 794)	
Age, years	48.98 ± 0.49	52.21 ± 1.15	50.23 ± 0.88	48.29 ± 0.78	47.05 ± 0.64	< 0.003
Sex, n (%)						0.2
Male	1146 (40.36)	219 (35.37)	266 (38.89)	318 (43.65)	343 (41.33)	
Female	1585 (59.64)	348 (64.63)	386 (61.11)	400 (56.35)	451 (58.67)	
Race, n (%)						< 0.001
Non-Hispanic White	1684 (80.91)	326 (76.62)	385 (78.67)	430 (79.97)	543 (85.27)	
Non-Hispanic Black	442 (7.13)	97 (9.04)	126 (9.34)	121 (7)	98 (4.80)	
Hispanic	75 (2.91)	15 (2.58)	17 (3.23)	25 (3.68)	18 (2.28)	
Mexican American	436 (4.88)	104 (5.88)	103 (5.04)	120 (5.52)	109 (3.78)	
Other race	94 (4.17)	25 (5.88)	21 (3.73)	22 (3.82)	26 (3.87)	
Education, n (%)						0.2
Less than high school	547 (12.14)	130 (14.56)	115 (10.63)	150 (12.85)	152 (11.38)	
High school graduate	613 (23.87)	124 (23.44)	161 (25.48)	148 (21.06)	180 (25.16)	
Some college or AA degree	733 (31.02)	143 (28.39)	164 (28.17)	197 (33.48)	229 (32.33)	
College graduate or above	687 (32.96)	139 (33.53)	171 (35.72)	180 (32.60)	197 (31.13)	
Poverty income ratio, n (%)						0.045
< 130%	576 (13.35)	139 (17.14)	125 (12.26)	144 (12.08)	168 (13.18)	
≥ 130%	2033 (86.65)	397 (82.86)	493 (87.74)	555 (87.92)	588 (86.82)	
Physical activity, n(%)						0.12
Inactive	73 (3.83)	11 (2.45)	13 (2.36)	18 (3.43)	31 (5.93)	
Insufficiently active	869 (46.97)	174 (42.50)	219 (49.87)	236 (47.12)	240 (47.16)	
Sufficiently active	873 (49.20)	193 (55.05)	203 (44.77)	241 (49.44)	236 (46.91)	
History of hypertension, n (%)						0.6
No	1421 (56.41)	385 (53.48)	484 (58.17)	552 (56.82)	552 (56.82)	
Yes	1309 (43.59)	398 (46.52)	424 (41.83)	487 (43.18)	487 (43.18)	
Diuretic use, n (%)						0.8
No	2231 (84.26)	622 (82.74)	761 (85.36)	848 (84.34)	848 (84.34)	
Yes	500 (15.74)	161 (17.26)	147 (14.64)	192 (15.65)	192 (15.65)	
BMI, kg/m^2^	29.70 ± 0.20	28.32 ± 0.41	29.19 ± 0.39	29.96 ± 0.40	30.53 ± 0.33	0.002
Na, mmol/L	139.07 ± 0.09	138.98 ± 0.13	139.05 ± 0.13	139.06 ± 0.10	139.15 ± 0.11	> 0.9
K, mmol/L	4.00 ± 0.01	4.01 ± 0.02	4.02 ± 0.02	3.99 ± 0.02	4.00 ± 0.01	0.6
Cl, mmol/L	103.58 ± 0.01	103.30 ± 0.12	103.50 ± 0.14	103.64 ± 0.12	103.74 ± 0.11	0.43
UA, mmol/L	319.37 ± 2.09	309.66 ± 4.41	320.50 ± 4.55	323.62 ± 3.42	320.26 ± 3.00	0.11
Osmolality, mmol/kg	277.83 ± 0.19	277.75 ± 0.28	277.76 ± 0.31	277.87 ± 0.20	277.89 ± 0.23	> 0.9
eGFR, mL/min/1.73m^2^	88.47 ± 0.67	86.00 ± 1.39	86.93 ± 1.16	88.93 ± 0.87	90.39 ± 0.84	0.004
Diet soft drink intake, servings/day	0.96 ± 0.04	0.03 ± 0.001	0.15 ± 0.002	0.53 ± 0.009	2.28 ± 0.059	< 0.001
Dietary sodium intake, mg/day	3355.30 ± 45.20	3082.50 ± 88.27	3168.11 ± 86.75	3383.81 ± 61.26	3592.58 ± 73.36	< 0.001
Total moisture intake, g/day	2437.30 ± 62.67	2317.97 ± 90.58	2302.84 ± 81.60	2431.92 ± 74.14	2588.92 ± 76.52	< 0.001
Total energy intake, kcal/d	1950.90 ± 19.45	1912.31 ± 43.72	1860.23 ± 43.30	2002.46 ± 39.52	1990.84 ± 32.71	0.061

*Abbreviations*: *BMI* Body mass index, *eGFR* Estimated glomerular filtration rate, *UA* Uric acid

^1^Values are presented as mean ± SE for continuous variables and unweighted numbers (weighted %) for categorical variables

^2^*P*-values are for the difference between the four diet soft drink quantiles. For the determination of *p*-values, svy-t-tests were used for continuous variables, and svy-wald-tests were used for categorical variables

^a^Data are presented incorporating sample weights and adjusted for clusters and strata of the complex sample design of the National Health and Nutrition Examination Survey (2003–2004 cycle and 2005–2006 cycle)

### HFCS sweetened soft drink and serum sodium

We used survey-weighted generalized linear regression models to examine the relationship between HFCS sweetened drink intake, as a continuous and categorical variable, and serum sodium. Overall, there was a step-wise increase in serum sodium concentration with increasing consumption of HFCS sweetened beverages (Table 3). After adjusting for age, sex, and race (model I), compared with the quantile1 group, participants with quartile 2, quartile 3, and quartile 4 had a significantly higher level of serum sodium. Compared with the quantile1 group, serum sodium was 0.32 mmol/L (0.09, 0.53, *p* = 0.009) higher in the quantile4 group. After further adjusting for education, poverty income ratio, physical activity, total energy, and history of hypertension (model II), the results were roughly consistent with model I. After further adjusting for BMI, diuretics use, serum potassium, serum chloride, osmolality, eGFR, and intake of dietary sodium, total moisture (model III), a step-wise relationship was found between HFCS consumption quantiles and serum sodium. Compared with the quantile1 group, serum sodium was 0.16 mmol/L higher in the quantile2 group and this effect approached statistical significance (Q2 vs. Q1, *p* = 0.051). Compared with the quantile1 group, serum sodium was 0.19 mmol/L higher in the quantile3 group (Q3 vs. Q1, *p* = 0.038) and 0.21 mmol/L higher in the quantile4 group (Q4 vs. Q1, *p* = 0.014), and all were statistically significant. After further adjustment of serum uric acid (model IV), the results remain unchanged. Even moderate HFCS sweetened soft drink intake was associated with an elevated serum sodium level. The results of the survey-weighted generalized linear regression were similar to those of HFCS sweetened drink intake as a continuous variable. Serum sodium level was 0.05 mmol/L higher with per 1 serving/day increase in HFCS sweetened drink intake (0.00, 0.11, *p* = 0.049).

**Table 3:** Relationship between HFCS sweetened drink, diet soft drinks intake and serum sodium in different models

Intake, servings/day	Model I		Model II		Model III		Model IV	
	β (95%CI)^**a**^	***p***-value	β (95%CI)^**a**^	***p***-value	β (95%CI)^**a**^	***p***-value	β (95%CI)^**a**^	***p***-value
**HFCS Sweetened drinks**	**(*n*** **= 5396)**		**(*n*** **= 2547)**		**(*n*** **= 2547)**		**(*n*** **= 2547)**	
Per 1 serving increment	0.05 (− 0.02,0.11)	0.185	0.06 (−0.06,0.18)	0.281	0.05 (0.00,0.11)	0.044	0.05 (0.00,0.11)	0.049
Quantiles								
Q1	Reference		Reference		Reference		Reference	
Q2	0.32 (0.17,0.58)	0.003	0.39 (0.06,0.74)	0.026	0.16 (0.00,0.33)	0.051	0.16 (−0.02,0.34)	0.065
Q3	0.22 (− 0.02,0.42)	0.070	0.19 (− 0.16,0.53)	0.255	0.19 (0.02,0.37)	0.038	0.19 (0.00,0.38)	0.049
Q4	0.32 (0.09,0.53)	0.009	0.34 (−0.08,0.72)	0.093	0.21 (0.07,0.36)	0.014	0.21 (0.05,0.37)	0.020
*P* for trend		0.105		0.139		0.047		0.051
**Diet soft drinks**	**(*n*** **= 2371)**		**(*n*** **= 1535)**		**(*n*** **= 1535)**		**(*n*** **= 1535)**	
Per 1 serving increment	0.06 (−0.02,0.14)	0.138	0.06 (−0.05,0.18)	0.279	0.00 (−0.04,0.04)	0.956	0.00 (−0.04,0.05)	0.967
Quantiles								
Q1	Reference		Reference		Reference		Reference	
Q2	0.08 (−0.19,0.35)	0.561	0.10 (−0.21,0.42)	0.492	0.06 (−0.12,0.23)	0.459	0.05 (−0.14,0.24)	0.513
Q3	0.11 (−0.18,0.40)	0.467	0.27 (−0.08,0.63)	0.118	0.06 (−0.10,0.22)	0.410	0.05(−0.12,0.23)	0.448
Q4	0.23 (−0.04,0.50)	0.116	0.26 (−0.15,0.67)	0.191	0.06 (−0.14,0.25)	0.498	0.05 (−0.15,0.26)	0.530
*P* for trend		0.093		0.321		0.759		0.760

Model I adjusted for age, sex and race

Model II adjusted for Model I + education, poverty income ratio, physical activity, total energy, history of hypertension

Model III adjusted for Model II + BMI, diuretics use, serum potassium, serum chloride, osmolality, eGFR, and intake of dietary sodium, total moisture

Model IV adjusted for Model III + serum uric acid

*Abbreviations*: *CI* Confidence interval, *HFCS* High fructose corn syrup

^a^Values are β coefficients (95% CIs)

### Diet soft drink intake and serum sodium

No significant relationship was found between diet soft drink consumption and serum sodium after adjustment of age, sex, and race (Q4 vs. Q1, *p* = 0.116) (Table 3). In the fully adjusted model, there was also no association between diet soft drink consumption and serum sodium both as a continuous (*p* = 0.967) and a categorical variable (Q4 vs. Q1, *p* = 0.530).

### Subgroup analysis

**Fig. 2:**
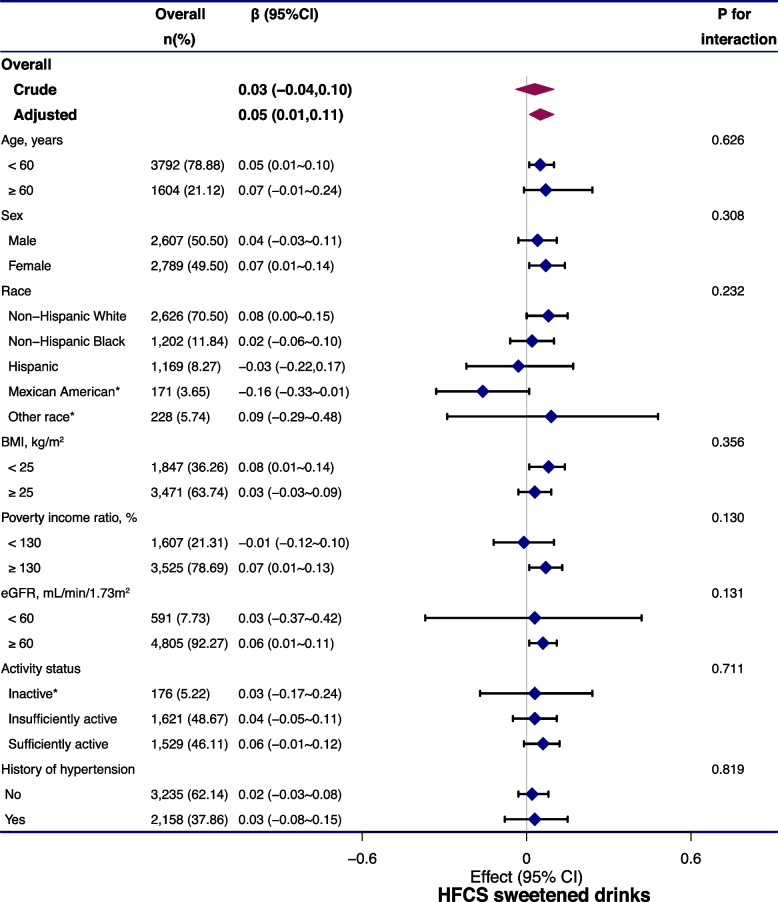
Stratified analysis of the association between HFCS sweetened drink intake and serum sodium, adjusted, if not stratified, for age, sex, race, education, poverty income ratio, physical activity, history of hypertension, diuretics use, BMI, serum potassium, serum chloride, serum uric acid, osmolality, eGFR, and intake of dietary sodium, total moisture and total energy; * values were not adjusted due to small sample size. Abbreviations: BMI: BMI, body mass index; CI, confidence interval; eGFR: estimated glomerular filtration rate; HFCS, high frucose corn syrup

Stratified analyses were conducted to examine whether the positive relationship between HFCS sweetened drink intake and serum sodium varied by age, sex, race, BMI, poverty income ratio, eGFR, activity status, and history of hypertension (Fig. 2). Numerically higher serum sodium concentrations were detected in age < 60 years, females, Non-Hispanic Whites, BMI < 25 kg/m^2^, eGFR≥60 mL/min/1.73m^2^, good economic status, and insufficiently active status. None of the variables, including age (*p* for interaction = 0.626), sex (*p* for interaction = 0.072), race (*p* for interaction = 0.170), BMI (*p* for interaction = 0.525), eGFR (*p* for interaction = 0.540), poverty income ratio (*p* for interaction = 0.294), activity status (*p* for interaction = 0.947), history of hypertension (*p* for interaction = 0.819) significantly modified the association between HFCS soft drink intake and serum sodium (Fig. 2).

**Fig. 3:**
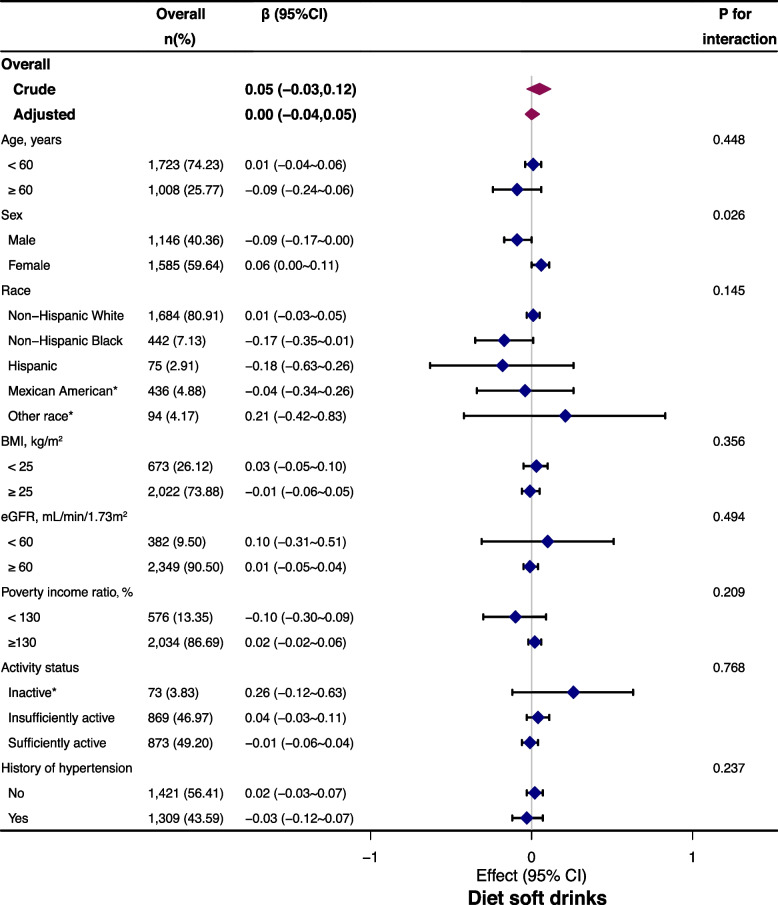
Stratified analysis of the association between diet soft drink intake and serum sodium, adjusted, if not stratified, for age, sex, race, education, poverty income ratio, physical activity, history of hypertension, diuretics use, BMI, serum potassium, serum chloride, serum uric acid, osmolality, eGFR, and intake of dietary sodium, total moisture and total energy. * values were not adjusted due to small sample size. Abbreviations: BMI: BMI, body mass index; CI, confidence interval; eGFR: estimated glomerular filtration rate

Stratified analyses were also conducted to examine whether the negative relationship between diet soft drink intake and serum sodium varied by age, sex, race, BMI, poverty income ratio, eGFR, and activity status (Fig. 3). Higher serum sodium concentrations were detected in females (males vs. females; *p* for interaction = 0.026). Meanwhile, numerically higher serum sodium concentrations were detected in age < 60 years, other race group, BMI < 25 kg/m2, eGFR< 60 mL/min/1.73 m2, good economic status, and inactive status. None of the variables, including age, race, BMI, eGFR, poverty income ratio, activity status, or history of hypertension significantly modified the association between diet soft drink intake and serum sodium (all p for interactions > 0.05; Fig. 3).

## Discussion

In this nationally representative sample of adults from the United States, a linear relationship was found between HFCS sweetened drink intake and serum sodium concentration, after adjustment for age, sex, race, education, poverty income ratio, physical activity, history of hypertension, diuretics use, BMI, serum potassium, serum chloride, osmolality, eGFR, and intakes of dietary sodium, total moisture, total energy. In consumers of HFCS sweetened soft drinks, each additional 1 serving/day intake of HFCS sweetened drinks was associated with 0.05 mmol/L higher serum sodium adjusted for confounders. Subgroup analyses will help in better understanding the trend of HFCS sweetened drink intake and serum sodium concentration in different populations. Higher serum sodium concentrations were detected in age ≥ 60 years, females, Non-Hispanic Whites, BMI < 25 kg/m^2^, eGFR ≥60 mL/min/1.73m^2^. High fructose corn syrup sweetened drink consumption is associated with serum sodium levels in this nationally representative sample of adults from the United States, but diet soft drink consumption is not. These results are consistent with our hypothesis.

Accordingly, we adjusted total moisture intake, dietary sodium intake, total energy intake, activity status, and other covariates, such as serum BMI, potassium, serum chloride, serum uric acid, eGFR, history of hypertension, diuretics use, and demographic variables that may affect serum sodium. A linear relationship was found between HFCS sweetened drink intake and serum sodium concentration after adjusting for confounding.

Industry sponsored studies have promoted the point of view that HFCS is chemically similar to sucrose [51]. As a consequence, previous studies might be focused on the metabolic consequences of fructose overconsumption and biochemical mechanisms that link fructose overconsumption with cardiometabolic risk factors [24, 26]. Results of our study revealed that the association between HFCS sweetened soft drink intake and serum sodium is independent of demographic (age, sex race, education, poverty income ratio), lifestyle (physical activity status), family history (history of hypertension), and dietary factors (serum potassium and chloride, osmolality, dietary sodium, total moisture). It is also independent of serum uric acid, which indicated that HFCS sweetened soft drinks might also affect hypertension through pre-metabolic mechanisms. Therefore, our findings suggested that mechanisms that link HFCS sweetened soft drink intake and hypertension appear independent of these risk factors. The intestine’s capacity to absorb fructose is saturable, and a healthy adult’s ability to absorb free fructose ranges from less than 5 g to more than 50 g [52]. Free fructose is associated with fructose malabsorption [52]. A lower level of intestinal fructose absorption is associated with a higher level of luminal fructose concentration [14]. Unabsorbed fructose affects bacterial load and modifies the composition of the gut microbiome [53]. Gut microbiota dysbiosis [53], gut formation of advanced glycation end-products [14], and the altered ratio of SCFA [19] may drive the onset of hypertension. Our findings are consistent with the research which shows that fructose induced changes to the gut microbiome are linked to hypertension.

In our study, no significant relationship was found between diet soft drink consumption and serum sodium after adjustment of potential confounders. Kim et al. reported that high artificially-sweetened soft drink consumption is associated with an increased risk of hypertension [9]. Interestingly, the positive relationship disappeared after additional adjustment for BMI. Meanwhile, a number of studies reported positive associations between diet soft drink consumption and the risk of metabolic diseases such as obesity and metabolic syndrome [54, 55]. However, Residual confounding factors such as dietary behaviors, lifestyle factors, or demographic characteristics may affect the associations [36].

There are some strengths to our study as follows: (1) This study is an observational study and some unmeasured confounding bias exists. We used strict statistical adjustment to minimize residual confounders (2) We handled the target independent variable as both a continuous variable and categorically. Such an approach can reduce the contingency of data analysis and enhance the robustness of data interpretation. (3) The effect modifier factor analysis takes full advantage of the data and yield stable conclusion in different subgroups in the present study. (4) In NHANES 2004–2004 and 2005–2006, over of 50% participants reported consuming HFCS drinks and our analyses used a large nationally representative sample of adults from the United States. Our results regarding the association between HFCS drink consumption and serum sodium levels are broadly generalizable to the US population.

There are some limitations in the present study which include: (1) Only approximately one-third of the participants reported consuming diet soft drinks. Our results regarding association between diet soft drink consumption and serum levels may be subject to selection bias. (2) Another main limitation of our study is its cross-sectional design, which prevents us from making a causal association, and is also vulnerable to recall bias. (3) The serum sodium levels were only detected at a single time point, not allowing for an evaluation of the dynamic changes.

## Conclusions

There was a step-wise increase in serum sodium concentration with increasing consumption of HFCS sweetened beverages. Even moderate HFCS sweetened soft drink intake was associated with an elevated serum sodium level - a risk factor for hypertension.

## Data Availability

The datasets generated and analysed during the current study are available in the NHANES repository, https://www.cdc.gov/nchs/nhanes/index.htm.

## Abbreviations

BMI: Body mass index
CKD-EPI: Chronic Kidney Disease Epidemiology Collaboration
Cl: Blood chloride
eGFR: Estimated Glomerular filtration rate
HFCS: High fructose corn syrup
MEC: Mobile examination centers
METs: Metabolic equivalents
NHANES: National Health and Nutrition Examination Survey
SCFA: Short-chain fatty acids
